# Risk Factors Associated with Upper Extremity Musculoskeletal Disorders among Barbers in Gondar Town, Northwest Ethiopia, 2018: A Cross-Sectional Study

**DOI:** 10.1155/2019/6984719

**Published:** 2019-04-03

**Authors:** Tesfaye Hambisa Mekonnen, Giziew Abere, Shalema Wedajo Olkeba

**Affiliations:** ^1^Department of Environmental and Occupational Health and Safety, Institute of Public Health, College of Medicine and Health Sciences, University of Gondar, Gondar, Ethiopia; ^2^North Shoa Zone Health Office, Oromia Region, Ethiopia

## Abstract

**Background:**

Work-related upper extremity musculoskeletal disorders (WUEDs) often present remarkable health and economic burdens on society. Occupational barbers are usually neglected both in research and policy actions, mainly in developing countries, and hence are likely subjected to the conditions. So far, information about factors that influence WUEDs among barbers in Ethiopia is inconclusive. Therefore, this study aimed to evaluate prevalence and factors associated with WUEDs among barbers in Gondar town, Ethiopia.

**Methods:**

We conducted a cross-sectional study from March to April 2018. A sample of 424 participants were recruited using systematic random sampling technique. A standardized Nordic Musculoskeletal Questionnaire was pretested and interviewer-administered for data collection. The data were analyzed by SPSS version 20 software. We set statistical significances at <0.05 *p* value with 95% confidence intervals (CI) and computed odds ratios to evaluate strength of associations.

**Results:**

The response rate was 98.3% (*N*=417). The mean age was 26.39 (SD + 4.805) years. The prevalence of upper extremity musculoskeletal disorders over the past 12 months was 56.8% (*N*=237). Upper back pain was observed in 38.8% (*N*=162) participants, whereas shoulder (27.1% (*N*=113)), neck pain and elbow/forearms (each 29.3% (*N*=122)), and wrists/hand disorders (32.4% (*N*=135)) were the common body sites indicated. Age (AOR: 2.614; 95% CI (1.287, 5.307)), alcohol use (AOR: 3.556; 95% CI (2.212, 5.717)), frequent standing (AOR: 1.536; 95% CI (1.006, 2.346)), physical exercises (AOR: 1.938; 95% CI (1.216, 3.089)), and low monthly salary (AOR: 3.125; 95% CI (1.157, 5.441)) were factors associated with work-related upper extremity disorders.

**Conclusions:**

Work-related upper extremity disorder is common among hairdressing professionals. Worksite health promotions targeted to lifestyle behaviors, like physical exercise and alcohol consumption require urgent public health actions in Ethiopia. Moreover, adaption of flexible work postures and proper management of workplace conditions related to aging workforces are also imperative to trace the complaints.

## 1. Introduction

Working as a barber is one of the precarious occupations with several workplace risk factors inherently associated with the profession [1]. A combination of exposure to various physical, chemical, ergonomic, psychosocial, and biological hazards among barbers is usually noticeable [2, 3]. Therefore, barbers are often subjected to various work-related disorders, like musculoskeletal (WRMSDs) disorders [4]. Work patterns that include fixed or constrained body positions, continual repetition of movements, force concentrated on small parts of the body, such as the hand or wrist, a pace of work that does not allow sufficient recovery between movements, vibration, and temperature incline to the development of WRMSDs [5–8].

Work-related musculoskeletal disorders are the major public health trepidations due to the rising demands of healthcare service utilization, temporary and permanent disability, and reduced quality of life associated with the conditions [7, 9]. Moreover, these disorders are contemporary occupational health problems, representing reduced productivity, absence from work, and escalating compensation premiums [9, 10]. For instance, in the United Kingdom (UK), an estimated 8.9 million work days were lost due to WRMSDs, constituting 35% of all days lost due to work-related ill health in 2016/17 [5]. Work-related upper limb disorders accounted for around 44% of days lost. A study done in Turkey also demonstrated that WRMSDs account for 34% of all work days lost due to work-related ill health [7].

Because of the nature of hazardous working conditions to which they are exposed, barbers are the potential high risk groups to musculoskeletal disorders, particularly to work-related upper extremity disorders (WUEDs) [2, 5, 11]. Hence, literature reveals that neck and shoulder pain affects between 6 and 76% of the working population annually [2]. A report in the United States (US) delineated that the prevalence of neck/shoulder disorders was 24% and distal upper extremity was 16% [12]. A study in Australia showed that 67.4% of the participants indicated neck, 46.3% shoulder, and 39.5% upper back pains in the past 12 months [13]. A systematic review conducted in the Netherlands illustrated that 12-month prevalence of WUEDs varies in the ranges of 2.3–41% [9]. The prevalence of 83.8% WUED in the neck has been found in one study [14], while 45% was found in a study in India, of which 35.69% of the total problems were related to neck, 17.44% to shoulder, 19.62% to arm and forearm, 16.08% to wrists, and 11.17% to hands.

The study in Iran [15] demonstrated that a magnitude of WUEDs was observed 55.9% in the neck and 43.8% shoulder.

Multiple risk factors determining the experience of WUEDs were previously researched. Sociodemographic characteristics, like sex, age, marital status, experience [7, 12, 16], workplace factors, like working hours, job tenure, type of activity (static and/or dynamic), work shift, safety training, working position, and rest breaks [14, 16–19], psychosocial factors including job satisfaction and stress [12, 14, 17, 20], behavioral styles, including alcohol consumption [21], physical activities [14, 20, 22] and body mass index [18, 23], and having a previous history of systemic illnesses [12, 24] were some of the potential risk factors which have been investigated to importantly predict the development of WUEDs.

The majority of studies addressing health problems, particularly work-related musculoskeletal disorders related to hairdressing professions, are usually restricted to the developed nations [25]. In the developing countries, including Ethiopia, informal sectors, such as occupational barbershops, are tremendously growing, generating employment opportunities, particularly for the poorest classes [26]. However, data on informal sectors, including barbershop occupations, are poorly documented, sometimes difficult to reach, and therefore, they are usually disregarded both in research and policy actions [27]. Consequently, these groups of working segments are often forced to work under aberrant and poor working conditions, which put them to various adverse health outcomes. Only few studies had been conducted in Ethiopia among barber professionals, particularly on the potential risks of HIV/IDS transmission [28] during regular practice and on the barbers' knowledge and practice towards occupational biological hazards [29].

Knowledge on the potential risk factors of upper extremity musculoskeletal disorders among barber professionals is, however, meager, and research is scant in Ethiopia. Therefore, the objective of this study was to investigate the prevalence and risk factors predisposing to WUEDs among barbers in Gondar town, Northwest Ethiopia.

## 2. Materials and Methods

### 2.1. Study Design and Period

A cross-sectional study was conducted from March to April 2018 to determine the prevalence and explore risk factors influencing upper extremity musculoskeletal disorders among barbers in Gondar town.

### 2.2. Study Setting and Area

This study was conducted among hairdressing professionals working in Gondar town, Northwest Ethiopia. The town is one of the tourist destination places found in Amhara regional state, Northwest Ethiopia, 747 km away from Addis Ababa, the capital city of the country. The town has 12°36′N latitude and 37°28′E longitude with an elevation of 2133 meters above the sea level. According to the 2007 Central Statistical Agency of Ethiopia (CSA), the town has a total population of 207,044 of whom 108,924 were women. During the data collection period, there were 1150 barbers in about 20 kebeles of the town.

### 2.3. Populations

All the barbers working in Gondar town were our source population. Barbers in the selected kebeles were the study population.

### 2.4. Inclusion and Exclusion Criteria

All the barbers who have worked for at least 12 months prior to the study period were included, while barbers who were on sick, annual, maternity, and family leaves were excluded.

### 2.5. Sample Size Determination

We used a single population proportion formula to calculate sample size required for the study. The following assumptions were considered to calculate the minimum sample size (*n*): *Z*=1.96 critical values with 95% CI (confidence intervals), *p*=50% (the proportion of upper extremity musculoskeletal disorders), and *d*=5% (margin of error). Since similar previous studies conducted among hairdressing professionals in Ethiopia are lacking, we applied the 50% assumptions for prevalence calculation. By inserting in the formula *n* = ((z^2^) (*p*) (1_*p*)) ÷ *d*^2^, *n* = ((1.96)^2^ (0.5) (1_0.5)) ÷ (0.05)^2^ = 384. Assuming a 10% for no responses, the final sample was 384 + 38.4 = 424.

### 2.6. Data Collection Tools and Procedures

Systematic random sampling technique was used to recruit eligible samples. An interviewer-administered questionnaire was applied to collect data. Prevalence of upper extremity musculoskeletal disorders was assessed using the questionnaire adapted from the standardized Nordic Musculoskeletal Questionnaire [30]. We evaluated perceived satisfaction of barbers with their jobs using the 10-item generic job satisfaction scale questionnaire [31]. This instrument is measured based on 5-Likert scale responses (strongly disagree = 1, disagree = 2, neutral = 3, agree = 4, and strongly agree = 5). The response scales were added together and summarized out of 50. We dichotomized the scores into a score of less than 32 = 0 (dissatisfied) and a score of 32 and above = 1 (satisfied) with their current jobs. We also assessed perceived job-related stress of the barbers by the 8-item workplace stress scale questionnaire [32]. This instrument is also measured based on 5-Likert scale responses (never = 1, rarely = 2, sometimes = 3, often = 4, and very often = 5) and added together to attain a summary score of 40. A final score was categorized into two with a score of less than 21 = 0 (not stressed) and a score of 21 and more = 1 (stressed). Both perceived job satisfaction and stress instruments had been used in a previous study conducted in Ethiopia [33]. The other detail contents of the questionnaire were developed from previous literature. The questionnaire was divided into four parts. The first part comprised sociodemographic characteristics, like sex, age, religion, educational status, marital status, monthly salary, and work experience. The second category covered organizational/workplace factors, including working hours per day, health and safety training, number of customers per day, pre and periodic medical examinations, shift work, work posture, and rest break. The third part of the questionnaire encompassed health and psychosocial factors, like previous history systemic illnesses, job satisfaction, and job stress. The behavioral style part covered the details of factors like physical exercise (yes/no), alcohol use (yes/no), smoking (yes/no), handedness (right/left), and body mass index (BMI) (weight divided by height square).

### 2.7. Data Quality Control

We gave priority for designing of appropriate data collection tools. The questionnaire was first developed in English and translated into local language “Amharic” and back to English by language experts to ensure consistency of the instrument. Eight environmental and occupational health and safety final year students in the College of Medicine and Health Sciences at the University of Gondar were involved in data collection after they took adequate training and orientation. Four well-experienced supervisors were recruited from Environmental and Occupational Health and Safety Department. The data collectors and supervisors had taken orientation on issues relating to clarity of the questionnaire, objectives of the study, confidentiality of information, and voluntary participation (informed consent) of the participants. The principal investigator supervised both the data collectors and supervisors. We also conducted a pretest on 18 samples in a kebele not involved in the final survey to test the validity and reliability of the data collection tool. We made few corrections to some misinterpretations and ambiguities, based on the pretest analysis.

### 2.8. Methods of Data Analysis

We manually cleaned the data for completeness, coded, and entered into EPI info version 7.1.5.2 and exported to SPSS version 20 software for analysis. Frequency distributions, percentages, means, and standard deviations were used for description of the results. The reliability of the standardized Nordic Musculoskeletal Questionnaire was tested using Cronbach's alpha, which and was found to be 0.67. According to Cronbach's alpha, the reliability of an instrument is tolerable in a given context at a cutoff point up to 0.65. The 10-item job satisfaction scale questionnaire was also examined for its reliability and Cronbach's Alpha was found to be 0.911. We also checked the 8-item job stress scale questionnaire, and Cronbach's alpha result was found as 0.798. The instruments were, therefore, tolerable for their consistency in repeating what had previously been measured using these tools. The associations between the dependent variable (upper extremities musculoskeletal disorders) and independent variables were examined by a binary logistic regression analysis. Accordingly, explanatory variables with a *p* value <0.2 in a bivariate analysis were exported to the multivariable logistic regression model to further investigate the potential effects of confounders. A forward variable selection method was used to drag variables into the multivariable logistic regression model. We checked goodness of the fit model by Hosmer and Lemeshow (*p* value >0.05). The odds ratios with 95% CI were calculated to evaluate strength of associations, and a cut off ≤0.05 *p* value was established to ascertain the significance of associations.

### 2.9. Operational Definition

*Upper extremity musculoskeletal disorder*: any type of upper body musculoskeletal disorders/pains, including neck, shoulder, arms and elbow, wrists, and hands that were experienced in the past 12 months.

*Body mass index (BMI)*:

  <18.5 = underweight
  18.5–24.99 = normal
  ≥25.0 = overweight/obesity

*Alcohol drinker*: an employee who drinks at least five drinks per week for men and two drinks per week for women for at least one year [34].

*Stressed worker*: the workplace stress scale score of 21 or above [32].

*Job-satisfied worker*: the generic job satisfaction scale score of 32 or above [31].

## 3. Results

### 3.1. Sociodemographic Characteristics

A total of 417 barbers were participated with a response rate of 98.3%. The majority, 86.8% (*N*=362), of the participants were males. Out of the total respondents, 85.9% (*N*=358) of them were those whose age were ≤30 and it ranged from 17 to 50 with a mean of 26.39 (SD ± 4.805) years. Regarding the educational level, 10.1% (*N*=42) of the respondents showed that they cannot read and write, 17% (*N*=71) reported they had attended primary education, 50.1% (*N*=209) had attended secondary education, and 22.8% (*N*=95) described that they had completed secondary and above education. The majority of the total participants, 60.4% (*N*=252), indicated they were single in marital status, 31.7% (*N*=132) were married, and 7.9% (*N*=33) were divorced (Table 1).

### 3.2. Behavioral and Psychosocial Factors

From among the respondents, about 35.7% (*N*=149) showed that they were alcohol users. A total of 42.2% (*N*=176) reported they were chat chewers, whereas 86.6% (*N*=361) were never smokers, 2.9% (*N*=12) were passive smokers, and 10.6% (*N*=44) were current smokers. Regarding physical exercises, 29.7% (*N*=124) elucidated that they performed physical exercise. The frequency of exercise was 10.1% (*N*=42) for barbers who execute exercise for 1-2 hours per day, 9.6% (*N*=40) for >2 hours per day, 5.8% (*N*=24) 1–3 times per week, and 4.3% (*N*=18) every day. Thirty-one percent (*N*=132) of the barbers clarified they had had systemic illnesses. Regarding perceived job satisfaction, only 33.8% (*N*=141) explained they were satisfied (job satisfaction score ≥32) with their jobs and 13.4% (*N*=56) represented they were stressed due to their jobs.

### 3.3. Organizational/Workplace Factors

More than half of the respondents, 50.4% (*N*=210), showed they engaged in temporary employment pattern. About 74.1% (*N*=309) of the participants' working hours per day were found to be more than 8 hours. Only 5.3% (*N*=22) of the barbers reported that they had taken safety training. Eighty percent (*N*=334) of the barbers included in this study were self-employed, whereas the remaining employed by others. Regarding the payment method, 24.2% (*N*=101) described that their payment scheme was on hourly basis, 53.5% (*N*=223) per piece rate, and 22.3% (*N*=93) were monthly based. Forty-nine percent (*N*=205) showed that they used rest breaks at their workplaces. The respondents also presented that 7.7% (*N*=32) of them spent 1–5 hours per day, 26.4% (*N*=110) spent 6–10 hours per day, and 65.9% (*N*=275) of them spent >6 hours per day standing at work.

### 3.4. Prevalence of Upper Extremity Musculoskeletal Disorders

The prevalence of upper extremity disorder in the past 12 months was 56.8% (*N*=237; 95% CI: (52.3, 61.4)). More than 48.9% (*N*=116) of the indicated complaints were observed in more than a single body site (comorbid). The upper body sites represented with the symptoms include upper back, 38.8% (*N*=162), shoulder, 27.1% (*N*=113), neck and elbow/forearms, each 29.3% (*N*=122), and wrists/hand 32.4% (*N*=135). About 55% (*N*=67) of the respondents with neck pains explained that they had experienced it in the last 7 days and 68.9% (*N*=84) were prevented from their work due to it. Out of the total respondents with shoulder pains, 86.7% (*N*=98) of them marked that they were prevented from their activities and 71.7% (*N*=81) reported that they experienced pain in the last 7 days. Moreover, among the complaints of elbow/forearms, 51.6% (*N*=63) of them demonstrated that they had been experiencing them in the last 7 days, whereas 33.6% (*N*=41) of them indicated they were prevented from activities because of their pain.

### 3.5. Risk Factors of Upper Extremity Musculoskeletal Disorders

In a bivariate analysis, predictor variables including age, monthly salary, working hours, work experience, health and safety training, alcohol drinking, working posture, exercise, payment methods, job satisfaction, and rest breaks were explored to substantially influence work-related upper body disorders. After controlling for confounding variables in a multivariable logistic regression model, only age, monthly salary, alcohol drinking, exercise, and working posture remained as the major contributors of upper extremity disorders.

Accordingly, participants whose ages were >30 years old were 2.61 times higher at risk to develop upper extremity disorders compared to the participants whose ages were ≤30 years old (AOR: 2.61; 95% CI (1.287, 5.307)). The respondents who indicated to consume alcohol everyday experience upper extremity musculoskeletal disorders 3.56 times more likely than those who indicated to consume alcohol once a week/occasionally (AOR: 3.56; 95% CI (2.212, 5.717)). Moreover, the odds of upper extremity disorders increased 1.54 times more likely among the participants whose work posture involved frequent standing than those whose work posture was optional/sitting and standing (AOR: 1.54; 95% CI (1.006, 2.346)). The likely occurrence of upper extremity disorders doubled among barbers who did not perform physical exercises than among those who performed them (AOR: 1.94; 95% CI (1.216, 3.089)). The chance of developing upper extremity disorders was 3.13 times higher for the participants who earned a low monthly salary (<1100 ETB) than those who earned a monthly salary of >1700 ETB (AOR: 3.13; 95% CI (1.157, 5.441)) (Table 2).

## 4. Discussion

This study employed a workplace-based cross-sectional study to evaluate the prevalence and risk factors associated with upper limb musculoskeletal disorders among barbers in Gondar town, Northwest Ethiopia. The prevalence of upper extremity musculoskeletal disorders among barbers in the previous 12 months was 56.8%. This finding was higher than the prevalence reported in Indonesia (22.4%) [21], Nigeria (31.5%) [35], India (45%) [3], and Malaysia (40.7%) [36]. The current investigation, however, found a lower prevalence of upper body disorders compared to the 82.5% prevalence reported from Iran [15] and 60% in the US [37]. Workplace health and safety services, illness and injury reporting procedures, data collection methods, and study participants could be the projected possible suggestions for these differences.

In the present study, out of the total indicated disorders of upper body regions, about 38.8% were related to upper back pain, 32.4% to wrists/hand disorders, 29.3% to the neck and elbow/forearm each, and 27.1% (*N*=113) to the shoulder. This result was relatively comparable with a study in Nigeria (34.7%) [38]. The comparable reports might be due to the reason that workplace illness reporting and management procedures relatively resemble in the developing countries. On the other hand, in the current study, the observed prevalence of upper back pain was higher as compared to the literature data from Indonesia (22.4%) [20] and Los Angeles (16%) [12], and lower when compared to the finding in India (73.44%) [17]. The discrepancies could be due to the difference in the data collection method and study participants.

This study found a lower prevalence of neck disorders compared to 35.69% prevalence of study finding in India [3], Brazil (47%) [39], Iran (55.9%) [19], Nigeria (46.3%) [40], and from the study in Ethiopia (51.7%) [24]. Among the participants, 27.1% of them had experienced shoulder pain in the past 12 months. This was higher than the prevalence of 17.44% reported in India [3] but lower than the 45% prevalence reported in Ethiopia [24], Brazil (49%) [39], Iran (43.8%) [19], and Nigeria (62.5%) [40]. The magnitude of musculoskeletal symptoms assessed in the elbow/forearm was 29.3%. The studies in Egypt [41] and India [3] had revealed lower findings (13.8% and 19.62%, respectively), compared to our result. The symptoms in wrists/hand were also evaluated and were found to be 32.4%, which was lower in magnitude when compared to 16.08% prevalence in India [3].

The multivariable regression model demonstrated that age was a markedly contributing factor for the development of upper extremity disorders. This result was in agreement with the other studies [2, 19, 38, 41, 42]. The probable explanation for these similarities is due to the fact that the biological/functional structures of human body, particularly those related to supportive structures, like muscles, joints, nerves, ligaments, and tendons, would tend to degenerate as age of the workers increases. This could likely induce to have a decreased functional capacity of workers. Similar explanations had been provided in other research findings [43, 44]. The other possible reason could be due to the effects of aging or a cumulative effect of workloads on the musculoskeletal system of workers through years of employment. Improvements in the modern human lifestyles would also likely increase workers' chance of extended stay in employment. resulting in the current aging workers dominating characteristics of workplaces.

Low monthly salary was investigated to significantly influence the experience of upper body musculoskeletal disorders. A study in Bangladesh supported this finding [42]. Workers with a lower salary are usually those who engage in the hazardous workplaces, who are not often covered by the national workers' health and safety protection programs, and those who are with a lower skill and knowledge. The combinations of all these situations could likely contribute to incur muscle injuries among barbers as they clear the floor for the exposure to these potential hazards. Moreover, in Ethiopia, barbers are usually professionals, many of whose wage system is based on piece-rate (performance based) payment schemes. Such types of wage systems possibly expose workers to increased physical efforts, perceived time pressure, and work pace demands as also outlined in the previous investigation [45]. Since employees with underearning usually endeavor to exhaustively utilize all their best possible capabilities to perform tasks that could generate and cover their financial constraints, they are often likely to be exposed to several health risk factors that may induce disorders in multiple body parts. Furthermore, it had been illustrated that earning a lower salary could have the potential to lower peoples' life standard and increase their health vulnerabilities [45]. However, a study conducted in Ethiopia among the workers of sewing machine operators had found inconsistent result [22]. A probable suggestion for this variation might be due to discrepancies in study population, nature of work environment, and the type of industry (informal versus formal).

Alcohol consumption is the other important predicting factor of upper body disorders. This result corroborated with previous studies [21, 40]. The plausible reason may be due to that alcohol drinking is one of the common health risk behavior that might deteriorate body's normal functional capacities and defense mechanisms. More possible explanation is that alcohol drinking might influence behavior of the people that often prohibits them from practicing a healthy lifestyle.

We examined that lack of physical exercise notably influenced likely occurrence of upper body musculoskeletal disorders. Recent studies have investigated similar findings [14, 22, 46, 47]. The possible suggestion for this finding is that performing a physical exercise on a regular basis might promote muscle strength, which helps retain it from getting easily injured on exposure to hazardous conditions. Moreover, engaging in physical exercise might positively influence the lifestyle of people. A systematic review conducted in Brazil [14] has confirmed the protective effects of physical exercise on musculoskeletal disorders. The study has also explained that doing any type of physical exercise for three times a week for 20 minutes promotes reduction of the pain of different body sites, including the upper extremity bodies. Further, another study explored that lack of exercise increases muscle stiffness and decreases their flexibility, making them susceptible to be damaged easily [47].

In this investigation, frequent standing was the other substantial risk factor that explained the increased probability of upper body disorders. This finding is supported by other studies [19, 40, 44, 48]. It could be explained that prolonged standing might impose stress on the body structures as a result of increased pressure in prolonged standing positions. A static/inflexible nature of working position for an extended period of time might also enhance muscle stiffness.

Of the greater importance of this study is that, to date, to the best of the investigators' understanding, the current study was the first to explore the prevalence and potential risk factors presenting to the experience of upper extremity disorders among barbers in Ethiopia. The situations contributing to upper body conditions were replicated, and the government and other stakeholders could benefit from for policy practice and implications. However, the self-report data collection method employed in this study might be the restriction, as recall bias and under-reporting might be anticipated from self-report. Moreover, it might be uncertain to generalize the findings of the study as the study was dealt with only a specific segment of the workforces. Therefore, further investigations which address adequate samples from various sectors with strong study designs, such as longitudinal studies, are often suggested.

## 5. Conclusions

Work-related upper extremity disorders are common among barbershop occupations. Age of the workers, low monthly salary, alcohol use, physical exercise, and standing for long time were identified to importantly affect WUEDs. Worksite health promotions targeted to lifestyle behavior, like physical exercise and alcohol consumptions requires policy attentions in Ethiopia.

Moreover, adaption of flexible work postures and proper management of workplace conditions related to aging work forces are imperative to curb the situation.

## Figures and Tables

**Table 1:** Sociodemographic characteristics of barbers, Gondar town, Ethiopia, 2018 (*N*=417).

Variables	Frequency	Percentage
Sex		
Male	362	86.8
Female	55	13.2

Age		
≤30 years	358	86.9
>30 years	59	14.1

Marital status		
Married	132	31.7
Single	252	60.4
Divorced	33	7.9

Educational status		
Cannot read and write	42	10.0
Primary education (1–8)	71	17.3
Secondary education (9–12)	209	50.1
Above secondary education	95	22.7

Monthly salary		
<1100 ETB	29	7.0
1101–1700 ETB	239	57.3
>1700 ETB	149	35.7

Religion		
Orthodox	344	82.5
Catholic	12	2.9
Protestant	31	7.4
Muslim	30	7.2

Experience		
≤5 years	300	71.9
>5 years	117	28.1

ETB = Ethiopian birr (currency); *N* = number.

**Table 2:** Factors associated with WUEDs among barbers, Northwest Ethiopia, 2018.

Variables (*N*=417)	Upper extremity disorders		Crude OR (95% CI)	Adjusted OR (95% CI)	*p* value
	Yes	No			
Age					
≤30 years	191	167		1	
>30 years	46	13	3.09 (1.616, 5.925)	2.61 (1.287, 5.307)	0.001^*∗∗*^

Monthly salary					
<1100 ETB	22	7	2.64 (1.063, 6.552)	3.13 (1.157, 6.441)	0.001^*∗∗*^
1101–1700 ETB	134	105	1.07 (0.710, 1.616)	1.54 (0.969, 2.436)	
>1700 ETB	81	68	1	1	

Work experience					
≤5 years	161	139	1		
>5 years	76	41	1.60 (1.028, 2.491)	0.80 (0.448, 1.416)	0.061^*∗*^

Working hours/day					
≤8 hours	51	57	1	1	
>8 hours	186	123	1.69 (1.087, 2.627)	1.35 (0.813, 2.226)	0.051^*∗*^

Safety training					
No	221	174	0.48 (0.183, 1.243)	0.45 (0.158, 1.279)	0.043^*∗*^
Yes	16	6	1	1	

Alcohol drinking					
No	123	145	1	1	
Yes	114	35	3.81 (2.435, 5.973)	3.74 (2.280, 5.717)	0.001^*∗∗*^

Physical exercise					
No	182	111	2.06 (1.344, 3.149)	1.94 (1.216, 3.089)	0.002^*∗∗*^
Yes	55	69	1	1	

Working posture					
Standing	126	77	1.52 (1.028, 2.243)	1.54 (1.006, 2.346)	0.103^*∗*^
Sitting	111	103	1	1	

Payment method					
Hourly	54	47	1	1	
Piece-rate work	120	103	1.01 (0.633, 1.635)	0.72 (0.342, 1.530)	
Monthly	63	30	1.83 (1.019, 3.280)	0.70 (0.349, 1.402)	0.110^*∗*^

Rest break					
No	113	99	0.75 (0.505, 1.100)	1.04 (0.613, 1.747)	0.022^*∗*^
Yes	124	81	1	1	

Job satisfaction					
Satisfied	89	52	1	1	
Not satisfied	148	128	1.48 (0.977, 2.244)	1.23 (0.702, 2.104)	0.140^*∗*^

^*∗*^Significant at a *p* value <0.2 in a bivariate analysis; ^*∗∗*^significant in multivariate analysis at a *p* value <0.05. ETB = Ethiopian birr; *N* = number; OR = odds ratio; CI = confidence interval.

## Data Availability

The data used to support the findings of this study are included within the article.

## Abbreviations

AOR: Adjusted odds ratio
BMI: Body mass index
BSc: Bachelor of Science
CI: Confidence interval
ETB: Ethiopian birr
km: Kilometer
MPH: Master of public health
MSc: Master of Science
OR: Odds ratio
SD: Standard deviation
UK: United Kingdom
US: United States of America
IF: Variance inflation factor
WUEDs: Work-related upper extremity disorders.
